# Intestinal *Clostridioides difficile* Can Cause Liver Injury through the Occurrence of Inflammation and Damage to Hepatocytes

**DOI:** 10.1155/2020/7929610

**Published:** 2020-09-12

**Authors:** Soomin Lee, Heeyoung Lee, Sejeong Kim, Jeeyeon Lee, Jimyeong Ha, Yukyung Choi, Hyemin Oh, Yujin Kim, Yewon Lee, Kyoung-Hee Choi, Yohan Yoon

**Affiliations:** ^1^Risk Analysis Research Center, Sookmyung Women's University, Seoul 04310, Republic of Korea; ^2^Food Standard Research Center, Korea Food Research Institute, Jeollabuk-do 55365, Republic of Korea; ^3^Department of Food & Nutrition, Dong-eui University, Busan 47340, Republic of Korea; ^4^Department of Food and Nutrition, Sookmyung Women's University, Seoul 04310, Republic of Korea; ^5^Department of Oral Microbiology, College of Dentistry, Wonkwang University, Iksan, Jeollabuk-do 54538, Republic of Korea

## Abstract

This study investigated if intestinal *Clostridioides difficile* (CD) causes liver injury. Four-week-old male C3H/HeN mice were treated with phosphate-buffered solution (control), CD, diethylnitrosamine (DEN) to induce liver injury with PBS (DEN+PBS), and DEN with CD (DEN+CD) for nine weeks. After sacrifice, livers and mesenteric lymph nodes (MLNs) were removed and bacterial translocation, transcriptomes, and proteins were analysed. CD was found in 20% of MLNs from the control and DEN+PBS groups, in 30% of MLNs from the CD group, and in 75% of MLNs from the DEN+CD groups, which had injured livers. Also, CD was detected in 50% of the livers in the DEN+CD group with CD-positive MLNs. Elevated *IL-1β*, *HB-EGF*, *EGFR*, *TGF-α*, *PCNA*, *DES*, *HMGB1*, and *CRP* expressions were observed in the CD and DEN+CD groups as compared to the control and DEN+PBS groups. Protein levels of IL-6 and HMGB1 were higher in the CD and DEN+CD groups than in the control and DEN+PBS groups. These results indicate that intestinal CD can initiate and aggravate liver injury, and the mechanism of pathogenesis for liver injury should be investigated in further studies.

## 1. Introduction

An enormous number of microorganisms, including bacteria, viruses, and archaea, inhabit the human body. The community of microorganisms that coexists peacefully has been called the microbiota, normal flora, or microflora [1]. Microorganisms that comprise the microbiota can colonise every surface of the body. The gastrointestinal tract is the most extensively colonised organ, housing approximately 70% of all microorganisms in the human body [2].

A balanced gut microbiota is critical to host health. However, overgrowth of pathogenic bacteria results in various diseases [3]. Changes in the gut microbiota can greatly impact the liver, because gut bacteria and their byproducts can enter the liver through the portal vein [4]. There are several reports on changes in gut microbiota associated with liver diseases such as nonalcoholic fatty liver disease, cirrhosis, alcoholic liver cirrhosis, and cirrhosis with encephalopathy [5–9].

An imbalance in the gut microbiota can be induced by exposure to a broad range of antibiotics. Several studies have shown the adverse effects of various antibiotics on the host gut microbiota in human subjects [10, 11] and animal models [12, 13]. Over the past few decades, both the incidence and severity of *Clostridioides difficile* infection (CDI) have increased dramatically worldwide [14]. In addition, several studies have shown an increase in CDI in patients with liver cirrhosis and liver transplant recipients [15, 16]. Patients with CDI and liver disease risk prolonged hospitalisation, immunosuppression, multiple comorbidities, chemotherapy, and the need for treatment with proton pump inhibitors [16]. In addition, the extent of microbiota perturbation in patients has been linked to the likelihood of developing recurrent CDI [17]. Thus, studies are needed to investigate whether *C. difficile* (CD) causes liver disease and aggravates liver disease when already present, in addition to its role in liver disease. To investigate the effect of *C. difficile* in intestine on liver injury, a mouse model is needed for preclinical studies.

Therefore, the objective of this study was to investigate the relationship of CD as an intestinal bacterium with the initiation and aggravation of liver injury using a mouse model.

## 2. Materials and Methods

### 2.1. Bacterial Inoculum Preparation

Toxigenic CD ATCC43594 was used in this study. CD was cultured in brain-heart infusion (BHI) medium with 10% fetal bovine serum (FBS), 0.2% glucose, and 1% sodium thioglycolate (ST) (BHI+FBS+G+ST) at 37°C for 48 h under anaerobic conditions established with Oxoid AnaeroGen (Thermo Fisher Scientific, Inc., Waltham, MA, USA). After incubation, the bacterial cells were harvested by centrifugation at 1,912 × g and 4°C for 15 min, washed twice, and resuspended in phosphate-buffered saline (PBS, pH 7.4; 0.2 g of KH_2_PO_4_, 1.5 g of Na_2_HPO_4_·7H_2_O, 8.0 g of NaCl, and 0.2 g of KCl in 1 L of distilled water).

### 2.2. Animal Procedures and DEN-Induced Liver Injury

**Figure 1:**
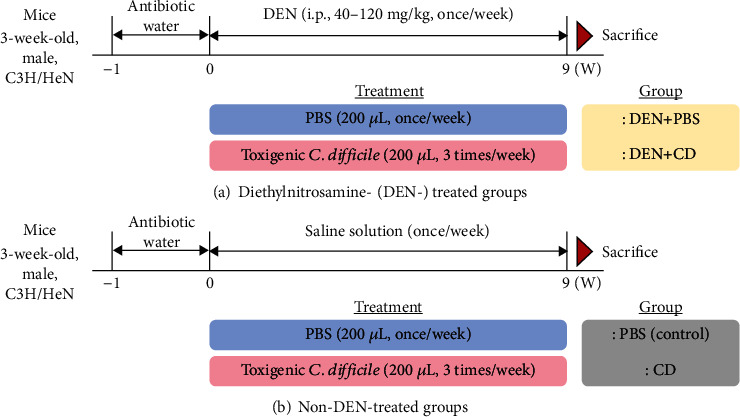
Experimental design in this study.

All animal experiments were approved by the Institutional Animal Care and Use Committee of Korea University, and the ethical approval number was KUIACUC-2017-62. The animal facility was a biosafety level 2 laboratory with individually ventilated cages under 12 : 12 light/dark cycles, and the bedding material was beta-chips. The animals were allocated to different experimental groups randomly, and three to four animals were placed in a cage. The experimental design is shown in Figure 1. Three-week-old male C3H/HeNCrljOri mice (Orient Bio, Inc., Seongnam, Gyeonggi, Korea) were given free access to chow diet and water, with a combination of clindamycin (100 mg/L) and streptomycin (5 g/L) supplied for five days in their drinking water to decrease commensal bacteria in their intestines and to enhance CD colonisation [18, 19]. The mice were then orally gavaged with (1) 200 *μ*L PBS (control; *n* = 5), (2) 200–300 *μ*L CD (at 4 log CFU/mL) three times a week for nine weeks (CD; *n* = 9), (3) weekly intraperitoneal injection (i.p.) of DEN (diethylnitrosamine; 40–120 mg/kg body weight) to induce liver injury and 200 *μ*L PBS oral gavage three times a week (DEN+PBS; *n* = 5), and (4) weekly DEN and CD treatment (DEN+CD; *n* = 8). The order in which the mice in the different experimental groups was treated was changed once a week to avoid bias effect.

During treatment, the activity and appearance of mice were monitored at 2–3-day intervals. After the mice were anesthetized by ether inhalation, they were euthanized by exsanguinating from vena cava, followed by removing livers and mesenteric lymph nodes (MLNs). To confirm the effect of DEN treatment for inducing liver injury, histological analysis was performed as follows. The mouse liver tissues were fixed in the 10% neutral buffered formalin and paraffin embedded, followed by staining with hematoxylin and eosin (H&E).

### 2.3. Bacterial Translocation Analysis

Detection of CD in an MLN culture was an indication of bacterial translocation from the gastrointestinal tract [20]. Hence, all MLNs were removed, and all were homogenised with 5 mm stainless beads (Qiagen) and TissueLyser LT (Qiagen) after resuspending MLNs in 1 mL 0.1% buffered peptone water (Difco, Becton, Dickinson and Company, Sparks, MD, USA). Each suspension was plated on *Clostridium difficile* moxalactam norfloxacin (CDMN) agar (Thermo Fisher Scientific, Inc.) to isolate CD, followed by anaerobic incubation at 37°C for 48 h. To detect CD in livers, a portion of each liver was homogenised using the same protocol as MLNs and plated on CDMN agar. After anaerobic incubation at 37°C for 48 h, colonies on plates were confirmed to be CD by PCR analysis. To identify CD, a primer set for *tpi* (CD species-specific gene) was used, and *tcdA* and *tcdB* (Table 1) were used for determining the toxin type of CD by multiplex PCR analysis using a Qiagen Multiplex PCR Kit (Qiagen) on a Rotor-Gene Q (Qiagen) with the following touch-down procedures: 95°C for 15 min; 94°C for 30 sec, decreased from 65°C to 55°C for an initial 11 cycles of 90 sec each; and then 40 cycles of 72°C for 30 sec, followed by a final extension at 72°C for 10 min. The PCR products were separated on 2% agarose gels, and the bands of PCR products were visualised in a LAS-3000 Imager (Fujifilm, Tokyo, Japan).

### 2.4. Transcriptome Analysis

Total RNA was extracted from livers and small intestines using an RNeasy Mini Kit (Qiagen) according to the manufacturer's instructions, and the RNA was quantified with a Take3 system in an Epoch Microplate Spectrophotometer (BioTek Instruments, Inc., Winooski, VT, USA).

For the quantitative real-time reverse transcription polymerase chain reaction (qRT-PCR) analysis, complementary DNA was synthesized from extracted total RNA using a QuantiTect Reverse Transcription Kit (Qiagen) according to the manufacturer's instructions. Primers used for qRT-PCR are listed in Table 1; primers from the QuantiTect Primer Assay (Qiagen) were used for tumour necrosis factor alpha (*TNF*-*α*) and interleukin-6 (*IL-6*). Glyceraldehyde-3-phosphate dehydrogenase (*GAPDH*) was used for normalisation of expression levels. qRT-PCR was performed on a Rotor-Gene Q instrument (Qiagen) using a Rotor-Gene SYBR Green PCR Kit (Qiagen) following the manufacturer's instructions. Relative fold changes were analysed using the −2^ΔΔ*C*_T_^ method. Relative expression levels are expressed as follows. One sample in the control group was designated the reference, and relative gene expression levels in other samples were calculated, followed by a calculation of the mean value and standard error of the control group. In treatment groups, the gene expression levels were measured against the level of the reference sample in the control group, and fold-changes in expression levels were expressed as means for the groups. According to a study by Sambanthamoorthy et al. [21], more than two-fold changes in gene expression were considered significant.

### 2.5. Immunoblotting

To investigate the expression level of protein related to cytokines in liver, at least three liver samples, randomly selected, per group were used for immunoblot analysis. To extract total proteins from livers and small intestines, frozen liver and small intestine tissues were prepared and homogenised in a PRO-PREP protein extraction solution (iNtRON Biotechnology, Inc.) for 25 min on ice, followed by centrifugation (13,000 rpm, 4°C, and 30 min). Proteins extracted from livers, and small intestines were quantified using a DC Protein Assay Kit I (Bio-Rad Laboratories, Inc., Hercules, CA, USA) according to the manufacturer's manuals. Forty micrograms of total protein from each sample was separated by sodium dodecyl sulfate polyacrylamide gel electrophoresis and transferred onto polyvinylidene difluoride membranes (GE Healthcare Life Sciences, Marlborough, MA, USA). These membranes were blocked with 5% skim milk (Sigma-Aldrich, St. Louis, MO, USA) for 1 h at room temperature. Immunoblots were performed with primary antibodies specific for IL-6 (sc-57315, 1 : 500; Santa Cruz Biotechnology, Inc., Dallas, TX, USA), HMGB1 (ab18256, 1 : 2500; Abcam, Cambridge, UK), PCNA (ab29, 1 : 2500; Abcam), and *β*-actin (sc-81178, 1 : 1000; Santa Cruz Biotechnology, Inc.). *β*-actin was used as a loading control. Horseradish peroxidase-conjugated anti-mouse IgG (sc-2005, 1 : 5000; Santa Cruz Biotechnology, Inc.) was used as a secondary antibody. To visualise reactive bands, membranes were developed with ECL Select Western blotting Detection Reagent (GE Healthcare Life Sciences), followed by chemiluminescence detection with an LAS-3000 Imager (Fujifilm). The intensity of immunoreactive bands was quantified using GelQuant software v. 2.7 (DNR Bio-Imaging Systems Ltd., Jerusalem, Israel).

### 2.6. Investigation of Intestinal Inflammation Effect on CD Translocation

An additional experiment (approval number: KUIACUC-2018-0043) was approved by the Institutional Animal Care and Use Committee of Korea University to prove if CD was translocated to MLNs and livers due to intestinal inflammation, which may be caused by DEN; DEN treatment was performed to induce liver injury. To induce intestinal inflammation in mice, five-week-old male C3H/HeN mice (Orient Bio, Inc.) were treated with 2% dextran sulfate sodium salt (DSS; MP Biomedicals, CA, USA) dissolved in drinking water for 7 days, followed by drinking water without DSS for the next 7 days. The mice were then orally injected with (1) 200 *μ*L PBS (control group; *n* = 8), (2) 200 *μ*L PBS and water with DSS (DSS+control group; *n* = 3), (3) 200 *μ*L *C. difficile* (a mixture of *C. difficile* strains ATCC43594 and ATCC BAA-1803) at 4 log CFU/mL (*C. difficile* group; *n* = 8), and (4) 200 *μ*L of the *C. difficile* mixture at 4 log CFU/mL and water with DSS (DSS+*C. difficile* group; *n* = 4) every day for 2 weeks. During treatment, the activity and appearance of mice were monitored at 2–3-day intervals. After the mice were anesthetized by ether inhalation, they were euthanized by exsanguinating from vena cava, followed by removing MLNs and livers. The MLNs and livers were homogenised with 5 mm stainless beads (Qiagen) and TissueLyser LT (Qiagen). From each tissue, DNA was extracted by a DNeasy Blood and Tissue Kit (Qiagen) according to the manufacturer's instructions. DNAs were used to detect CD by qRT-PCR, using the primer sets for 23S rRNA gene sequence (Table 1). qRT-PCR was performed in a Rotor-Gene Q instrument (Qiagen) using a Rotor-Gene SYBR Green PCR Kit (Qiagen) according to the manufacturer's instructions. To convert *C*_T_ values to log CFU/g, a standard curve was prepared. To produce the standard curve, CD culture was diluted to 1-7 log CFU/mL, the cell counts were enumerated on CDMN agar (37°C of incubation for 48 h), and qRT-PCR was also performed using the respective cultures. A linear equation was then applied to get the relationship between CD cell counts and *C*_T_ values.

### 2.7. Statistical Analysis

The statistical analysis was performed using SAS (version 9.2; SAS Institute, Inc., Cary, NC, USA). All data were analysed by the general linear model procedure, and the test of significance of least squares mean was performed using a pairwise t-test at *α*=0.05.

## 3. Results

### 3.1. Bacterial Translocation through MLN

**Figure 2:**
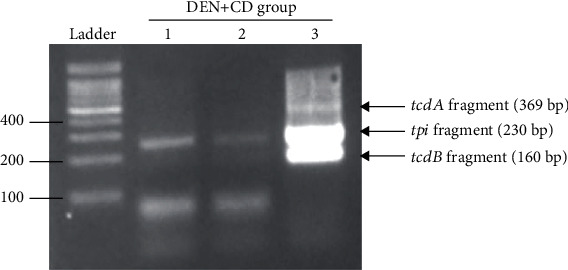
Multiplex PCR to detect *Clostridioides difficile* (CD) in livers of mice in the diethylnitrosamine (DEN)+CD group. CD colonies were confirmed from liver cultures in three of eight mice. Lane 1: *tpi*: +; *tcdA*: −; *tcdB*: −. Lane 2: *tpi*: +; *tcdA*: −; *tcdB*: −. Lane 3: *tpi*: +; *tcdA*: +; *tcdB*: +.

**Figure 3:**
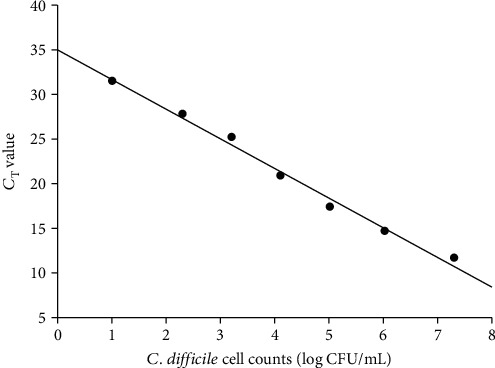
Standard curve between *Clostridioides difficile* cell counts (log CFU/g) and *C*_T_ values measured by qRT-PCR.

In our experiment, CD was detected in the MLN in three (33%) of nine mice inoculated with CD, whereas CD was detected in the MLN of only one (20%) of five mice inoculated with PBS (Table 2; Suppl. Figure 1). CD was detected in the MLN of one (20%) of five mice in the control and DEN+PBS groups, whereas CD was detected in three (33%) of nine mice in the CD group and in six (75%) of eight mice in the DEN+CD group (Table 2; Suppl. Figure 1). In addition, three liver samples from the DEN+CD group were CD-positive (Figure 2). The mice with these livers were identical with those of the mice with CD-positive MLNs. In both MLNs and livers, CD-positive rates were calculated based on the presence of *tpi* (species-specific DNA). After discontinuing antibiotic administration, the inhibited CD in intestine was recovered slowly; thus, both toxigenic and nontoxigenic CDs could be found. Even nontoxigenic CDs were found in more CD-treatment groups (CD and DEN+CD) than in non-CD treatment groups (control and DEN+PBS). It can be inferred that the injected CD weakened the gut barrier, causing more transmission of intestinal CDs (whether or not toxins exist), or toxigenic CDs administered may have been difficult for toxin genes to detect due to genetic variation or problems with DNA purification through the intestinal environment. To evaluate if CD is translocated from the intestine to the liver due to the intestinal inflammation, which may be induced by DEN treatment, we used DSS to induce intestinal inflammation without any damage to the liver. The result showed that no CD was detected in all liver tissues (data not shown). The additional experiment result showed that CD cell counts in the pure culture obtained by plating (*x* axis) and the corresponding *C*_T_ values obtained by qRT-PCR (*y* axis) showed a good correlation (*R*^2^ > 0.997) (Figure 3).

### 3.2. Change of Transcripts in Liver

**Figure 4:**
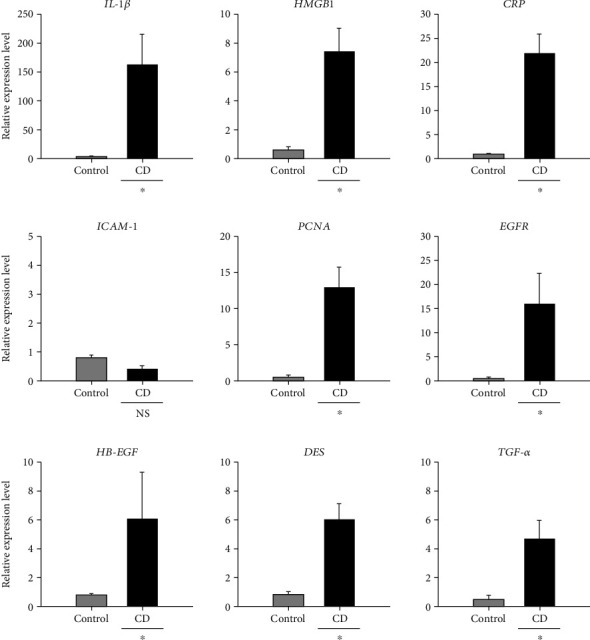
Relative gene expression levels in liver tissues from mice in the nondiethylnitrosamine- (DEN-) treated groups, the phosphate-buffered solution- (PBS-) treated group, and the *Clostridioides difficile*-only-treated (CD) group. All data are presented as the mean and standard error. ^∗^>2-fold changes were considered significant.

To evaluate the relative expression levels of genes associated with liver inflammation and injury, a qRT-PCR analysis was performed. The indicators of a proinflammatory response, liver injury, and hepatocarcinoma were investigated regarding TNF-*α*, IL-6, IL-1β, heparin-binding epidermal growth factor (HB-EGF), epidermal growth factor receptor (EGFR), transforming growth factor alpha (TGF-*α*), proliferative cell nuclear antigen (PCNA), desmin, intracellular adhesion molecule (ICAM-1), high mobility group box-1 (HMGB1), and C-reactive protein (CRP). Levels of *TNF*-*α* and *IL-6*, proinflammatory cytokines, were under the cut-off cycle threshold for this study. In non-DEN-treated groups, the relative gene expression of IL-1*β* was 35.85-fold higher in the CD group than in the control groups, indicating that a proinflammatory immune response occurred in livers with CD overgrowth in the intestinal tract. The *HMGB1* expression level was 11.38-fold higher in the CD group than in the control groups. In this study, in the non-DEN-treated groups, *CRP* was 12.37-fold higher (*p* < 0.05) in the CD group than in the control group (Figure 4). Gene expression of ICAM-1 in the CD group was 0.40-fold lower than that in the control group (Figure 4), indicating that inflammation may have occurred in the liver. In our study, *PCNA* levels were elevated 24.60-fold in the CD group relative to that in the control group (Figure 4), indicating that regeneration of hepatocytes might occur in the CD group. In Figure 4, *EGFR* levels in the CD group were 29.60-fold higher than those in the control group, indicating that cell regeneration and transformation might occur in the liver. *HB-EGF* expression levels were 3.54-fold higher in the CD group than in the control group (Figure 4). *DES* expression levels in the CD group were 4.07-fold higher than those in the control group (Figure 4). *TGF-α* expression in the CD group was 14.62-fold higher than that in the control group (Figure 4). These results indicate that intestinal CD may cause inflammatory responses in the liver and contribute to a liver cancer microenvironment.

**Figure 5:**
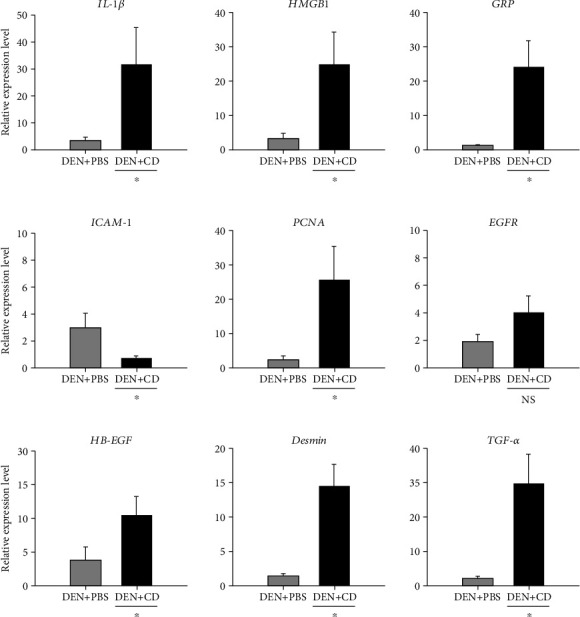
Relative gene expression levels in liver tissues from mice in the diethylnitrosamine- (DEN-) treated groups. DEN+phosphate-buffered solution (PBS) (*n* = 3): control group, intraperitoneally injected (i.p.) with 40–120 mg/kg DEN with PBS by oral gavage. DEN+*Clostridioides difficile* (CD) (*n* = 5): i.p., 40–120 mg/kg DEN with CD by oral gavage. All data are presented as the mean and standard error. ^∗^>2-fold changes were considered significant. NS: not significant.

In the DEN-treated groups of mice, among genes (*IL-1β*, *HMGB1*, and *CRP*) related to inflammation, *IL-1β* (6.40-fold), *HMGB1* (7.37-fold), and *CRP* (18.31-fold) expression levels were significantly higher (*p* < 0.05) in the DEN+CD group than in the DEN+PBS group (Figure 5), but among genes related to liver damage (*ICAM-1* and *DES*), only expression levels of *ICAM-1* were 0.25-fold lower in the DEN+CD group than in the DEN+PBS group (Figure 5). *Desmin* gene expression levels were 9.76-fold higher in the DEN+CD group than in the DEN+PBS group. The expression levels of hepatocyte regeneration-related genes (*PCNA*, *EGFR*, and *HB-EGF*) were 9.42-, 1.98-, and 3.20-fold higher in the DEN+CD group than in the DEN+PBS group (Figure 5). *TGF-α* was 14.88-fold higher in the DEN+CD group than in the DEN+PBS group (Figure 5).

### 3.3. Protein Level Related to Inflammation and Injury in Liver

**Figure 6:**
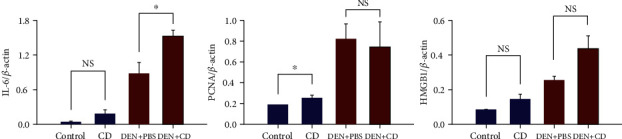
The immunoreactive intensity of liver proteins from mice in the phosphate-buffered solution- (PBS-) treated (control) group, the *Clostridioides difficile*-treated (CD) group, the diethylnitrosamine (DEN) and PBS-treated (DEN+PBS) group, and the DEN and CD-treated (DEN+CD) group. All data are presented as the mean and standard error. NS: not significant.

Relative protein levels were expressed as the levels of target protein to normalised protein (*β*-actin). In Figure 6, IL-6 protein levels, normalised to *β*-actin, were higher (*p* = 0.07) in the CD group (0.19 ± 0.05) than in the control group (0.04 ± 0.02) (Suppl. Figure 2A). The immunoreactivity of PCNA has been used to assess proliferative activity in normal, regenerative, and tumoral livers in humans and rodents [22]. PCNA protein levels were significantly elevated in the CD group (0.26 ± 0.02) (*p* < 0.05) relative to those in the control group (0.19 ± 0.00) (Figure 6; Suppl. Figure 2A). The relative protein levels of HMGB1, which plays a pivotal role in liver injury, were assessed in the control and CD-treated groups. The levels of HMGB1 protein in the PBS and CD groups were 0.08 ± 0.00 and 0.14 ± 0.02 (*p* = 0.10), respectively (Figure 6; Suppl. Figure 2A).

In the DEN-treated groups, significantly increased IL-6 was observed only in the DEN+CD group (1.53 ± 0.08) (*p* < 0.05), and not in the DEN+PBS group (0.89 ± 0.15) (Figure 6; Suppl. Figure 2B). The levels of PCNA in the DEN+CD (0.80 ± 0.25) and DEN+PBS (0.82 ± 0.12) groups were not significantly different (*p* > 0.05), but they were higher than those in the non-DEN-treated group (Figure 6; Suppl. Figure 2B). Even though the levels of HMGB1 in the DEN+CD (0.44 ± 0.06) and DEN+PBS (0.26 ± 0.02) groups were not significantly different (*p* = 0.09) (Figure 6; Suppl. Figure 2B), these levels were reasonably different.

## 4. Discussion

To investigate the impact of CD on liver damage, DEN was used to induce liver injury. Histological changes in DEN-treatment groups, compared to control, were observed (Suppl. Figure 3). Bacterial translocation occurs when bacteria colonising the gastrointestinal tract cross the mucous membrane and migrate to the mesenteric lymph nodes (MLNs), spleen, liver, and blood [20]. According to Garcia-Tsao et al. [20], CD-positive MLNs are an indication of bacterial translocation from intestines. In our study, we treated mice with DEN to induce liver injury, and the result showed that the rates of CD-positive MLNs were higher in the CD and DEN+CD groups than in the control and DEN+PBS groups, and the rates of CD-positive liver were higher in the DEN+CD group than in the control, DEN+PBS, and CD groups. However, DEN treatment may cause intestinal inflammation as Shirakami et al. [23] suggested, and it may help in the translocation of CD from the intestine to the liver. Thus, we induced intestinal inflammation in the mice with DSS, but no CD was detected in all liver samples. These results indicate that bacterial translocation of CD can be accelerated through MLNs if CD colonises the intestinal lumen and that this translocation can be accelerated when the liver is injured.

In analysis of transcripts in livers, the gene expression levels of IL-1*β*, HMGB1, and CRP were analysed to investigate immune reactions. IL-1*β* is a proinflammatory cytokine mainly produced by macrophages [24]. It is also a potent myofibroblastic activator of hepatic stellate cells [25]. HMGB1 is a nuclear protein released from immune cells or injured nonimmune cells [26] and a critical mediator of various inflammatory responses to injury, infection, and inflammation [27]. CRP, secreted by the liver, is an acute-phase protein that can bind to a microbial capsular polysaccharide and that is involved in innate immune reactions against bacteria [28]. This protein is mainly regulated by IL-6 or IL-1*β* from hepatocytes [22], and in humans, CRP is the most widely studied marker of systemic inflammation. An association between CRP expression levels and liver disease, including nonalcoholic fatty liver disease (NAFLD), fibrosis, and hepatitis has been reported [29]. Indicators related to liver injury and damage including ICAM-1 and desmin were investigated. ICAM-1 is a member of the immunoglobulin superfamily, and it is expressed on various cell types, including epithelial cells, endothelial cells, and fibroblasts. ICAM-1 is overexpressed in response to proinflammatory cytokines such as TNF-*α* and IL-1 [30]. In addition, this molecule has been reported to be involved in leukocyte-mediated tissue injury [31, 32] and to bind to leukocytes after partial hepatectomy, inducing hepatocyte proliferation in response to the release of TNF-*α* and IL-6 [33]. Desmin is a smooth muscle protein composed of intermediate filaments, and it is regarded as a representative marker of hepatic stellate cells [34]. To determine the level of liver regeneration, *PCNA*, *EGFR*, and *HB-EGF* were used. PCNA has been found in the nuclei of cells of organisms from yeasts to animals. It regulates cell division, the cell cycle, and/or DNA replication [35]. Several studies [36, 37] have shown that expression of this protein is linked to proliferation or neoplastic transformation. Hepatocytes are quiescent in the normal adult liver, and the cells are renewed quite slowly [38]. However, cell regeneration can occur following injury, and cell proliferation is a critical component to a regenerative reaction. This reaction is also regarded as essential for the initiation of carcinoma [39]. Hepatocyte proliferation was found to be elevated in human cirrhotic livers, and patients with elevated cell proliferation in cirrhotic livers are at increased risk of developing hepatocellular carcinoma (HCC) [40]. EGF and its tyrosine kinase receptor, EGFR, have been suggested to play a critical role in liver regeneration and transformation [41, 42]. EGFR is highly increased in human cirrhosis cases [43]. HB-EGF is a member of the EGF family, and it is produced in various tissues, including the lung, brain, heart, and skeletal muscle [44]. It is associated with various physiological and pathological processes such as wound healing, development, atherosclerosis, and blastocyst and tumour formation [44]. TGF-*α* plays a vital role in hepatocarcinogenesis in humans and animals, and *TGF-α* expression is increased in hepatocellular carcinoma (HCC) tissues; furthermore, TGF-*α* has been reported to be linked to the differentiation of HCC cells [45]. The results of this study showed that the relative gene expressions of IL-1*β*, HMGB1, CRP, PCNA, EGFR, HB-EGF, desmin, and TGF-*α* were significantly higher in the CD-treated groups (CD and DEN+CD groups) than in the PBS groups (control and DEN+PBS groups), while the *ICAM-1* level in the CD group was not significantly different from that in control. In addition, these results became more obvious when mice were treated with DEN. Thus, CD overgrowth in intestines appears to promote inflammation in the liver, and this effect can be more deleterious to injured livers. Although histological changes were not observed within nine weeks, these transcriptional changes indicate the potential for liver damage in the long term.

In protein expression analysis using western blot assay, IL-6, a proinflammatory cytokine, and PCNA and HMGB1 related to liver injury, were examined. IL-6 and TNF-*α* are considered critical drivers of inflammation [46]. They are regarded as pathogenic markers, and their expression is associated with liver inflammation and fibrosis [47]. In addition, according to research by Streetz et al. [48], IL-6 induces the production of acute phase proteins in the liver and accelerates liver generation. In both the CD and DEN+CD groups, IL-6 expression levels in the liver were increased compared to the control and DEN+PBS groups, although the increase in IL-6 of the CD group was not significant (Figure 6). However, we observed the tendency of the proinflammatory cytokine level in liver when CD is overgrown in the intestines. The increase in PCNA protein expression was observed only in the CD group compared to the control. The levels between the DEN+CD and DEN+PBS groups were similar, whereas transcriptional expression of PCNA was elevated in the DEN+CD group than in the DEN+PBS group. Also, HMGB1 protein levels were not significantly different, but they were elevated in CD-treated groups. These results indicate that colonisation of CD in intestines can cause transcriptional changes, and consequently it may promote to produce proteins affecting inflammation and damage to hepatocytes.

## 5. Conclusions

In summary, the positive rates of CD in MLNs, an indicator of bacterial translocation, were higher for the CD-treated groups compared to the control groups. In transcriptome analysis, the expression of genes related to proinflammatory cytokine, liver injury, and hepatocellular carcinoma was elevated in the CD and DEN+CD groups compared to the control groups. Also, the protein levels in the liver related to proinflammatory cytokine or liver injury were increased in the CD and DEN+CD groups compared to the PBS and DEN+PBS groups. Although this study has limitations as to whether the effect of CD in the intestines of mice with liver injury can be relevant to humans due to the different intestinal environment between mice and humans, these results have implicated CD in the intestines as a cause of liver disease through inflammation, and liver injury can be aggravated by CD from the intestines.

## Figures and Tables

**Table 1:** Primers for detection of *Clostridioides difficile* and quantitative real-time reverse transcription polymerase chain reaction.

Gene	Sequence (5′ to 3′)	Tm (°C)	Reference
*tpi*	F: AAAGAAGCTACTAAGGGTACAAA	55-65; touch-down	[49]
	R: CATAATATTGGGTCTATTCCTAC		
*tcdA*	F: AGATTCCTATATTTACATGACAATAT		
	R: GTATCAGGCATAAAGTAATATACTTT		
*tcdB*	F: GGAAAAGAGAATGGTTTTATTAA		
	R: ATCTTTAGTTATAACTTTGACATCTTT		
*23S*	F: GGGAGCTTCCCATACGGGTTG	60	[44]
	R: TTGACTGCCTCAATGCTTGGGC		
*GAPDH*	F: TCCTGCACCACCAACTGCTTAG	55	[50]
	R: TGCTTCACCACCTTCTTGATGTC		
*IL-1β*	F: CTCCATGAGCTTTGTACAAGG	55	[51]
	R: TGCTGATGTACCAGTTGGGG		
*HB-EGF*	F: GAAAGCAGGATCGAGTGAGC	60	[52]
	R: CTTGCGGCTACTTGAACACA		
*EGFR*	F: GGCGTTGGAGGAAAAGAAAG	60	[52]
	R: TTCCCAAGGACCACTTCACA		
*DES*	F: AGCTCAAGTCATCGCCCTTC	60	[52]
	R: GCAGATCCCAACACCCTCTC		
*TGF-α*	F: CAGGGAGCAACACAAATGGA	60	[52]
	R: AGCCTCCAGCAGACCAGAAA		
*PCNA*	F: TTTGAGGCACGCCTGATCC	55	[53]
	R: GGAGACGTGAGACGAGTCCAT		
*ICAM-1*	F: TCGGAAGGGAGCCAAGTAACT	60	[54]
	R: GATCCTCCGAGCTGGCATT		
*CRP*	F: ATG GAG AAG CTA CTC TGG TGC	60	[55]
	R: ACA CAC AGT AAA GGT GTT CAG TG		
*HMGB1*	F: CTTCGGCCTTCTTCTTGTTCT	60	[27]
	R: GGCAGCTTTCTTCTCATAGGG		

**Table 2:** Positive cultures of *Clostridioides difficile* in mesenteric lymph nodes (MLNs).

Experimental group	*n*	CD-positive MLN culture	CD-positive percentage (%)
PBS	5	1	20
DEN+PBS	5	1	20
CD	9	3	33
DEN+CD	8	6	75

## Data Availability

The data in this study are available from the corresponding authors on reasonable request.

## Supplementary Materials

Supplementary Materials Supplementary Figure 1: gel bands for *Clostridioides difficile* (CD) in mesenteric lymph nodes (MLNs) of mice in the diethylnitrosamine (DEN)+CD and CD groups. Presumed CD colonies on *Clostridium difficile* moxalactam norfloxacin agar were confirmed with tpi (species-specific) bands. White numbers on the gels indicate the MLN sample of mice. Supplementary Figure 2: protein expressions of IL-6, PCNA, and HMGB1 in liver tissues of non-DEN treatment groups (A) and DEN-treatment groups (B). DEN: diethylnitrosamine. Supplementary Figure 3: histopathological changes of mice liver in the control and diethylnitrosamine (DEN) treatment groups (200). Hematoxylin and eosin- (H&E-) stained liver from control, DEN+PBS, and DEN+CD. Liver from control presented a healthy state. In DEN+PBS, degenerated hepatocyte (black arrow), hepatocellular necrosis (black triangle), karyomegaly (star), oval cell hyperplasia (white arrow), and cholestasis (white triangle) were found. In DEN+CD, degenerated hepatocyte (black arrow), hepatocellular necrosis (black triangle), oval cell hyperplasia (white arrow), and cholestasis (white triangle) were found. Each scale bar is indicated in the lower right corner with a black line and represents 50 *μ*m.
