# Supplementary material for: Intestinal Clostridioides difficile Can Cause Liver Injury through the Occurrence of Inflammation and Damage to Hepatocytes
Source: Biomed Res Int. 2020 Sep 12;2020:7929610. doi: 10.1155/2020/7929610 (PMC7503108; doi:10.1155/2020/7929610)
Supplement: Supplementary Materials — Supplementary Figure 1: gel bands for Clostridioides difficile (CD) in mesenteric lymph nodes (MLNs) of mice in the diethylnitrosamine (DEN)+CD and CD groups. Presumed CD colonies on Clostridium difficile moxalactam norfloxacin agar were confirmed with tpi (species-specific) bands. White numbers on the gels indicate the MLN sample of mice. Supplementary Figure 2: protein expressions of IL-6, PCNA, and HMGB1 in liver tissues of non-DEN treatment groups (A) and DEN-treatment groups (B). DEN: diethylnitrosamine. Supplementary Figure 3: histopathological changes of mice liver in the control and diethylnitrosamine (DEN) treatment groups (200). Hematoxylin and eosin- (H&E-) stained liver from control, DEN+PBS, and DEN+CD. Liver from control presented a healthy state. In DEN+PBS, degenerated hepatocyte (black arrow), hepatocellular necrosis (black triangle), karyomegaly (star), oval cell hyperplasia (white arrow), and cholestasis (white triangle) were found. In DEN+CD, degenerated hepatocyte (black arrow), hepatocellular necrosis (black triangle), oval cell hyperplasia (white arrow), and cholestasis (white triangle) were found. Each scale bar is indicated in the lower right corner with a black line and represents 50 μm. [file 7929610.f1.doc]

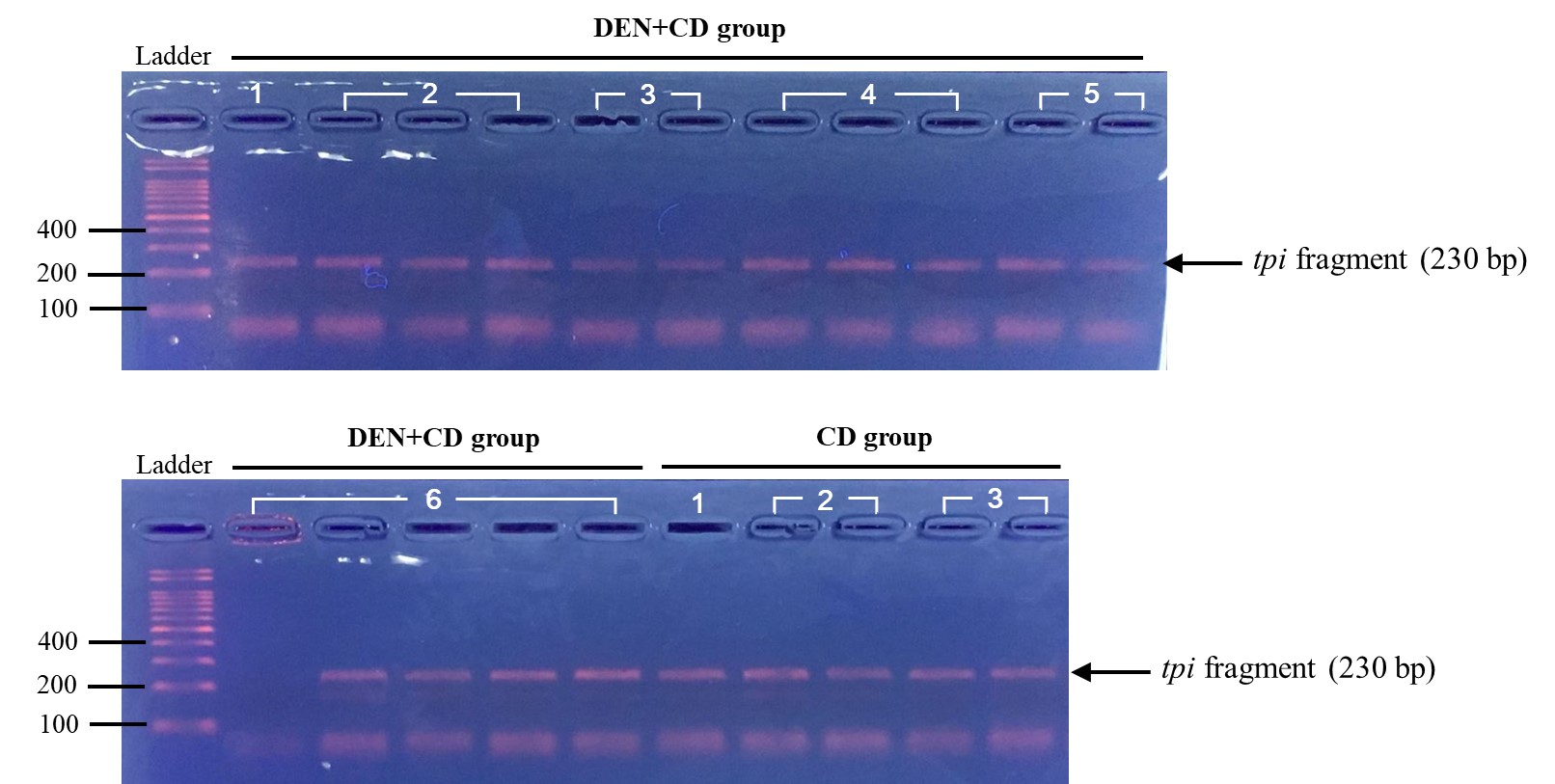


**Supplementary Figure 1.** Gel bands for *Clostridioides difficile* (CD) in mesenteric lymph nodes (MLNs) of mice in the diethylnitrosamine (DEN)+CD and CD groups. Presumed CD colonies on *Clostridium difficile* moxalactam norfloxacin agar were confirmed with *tpi* (species-specific) bands. White numbers on the gels indicates MLN sample of mice.

**A**


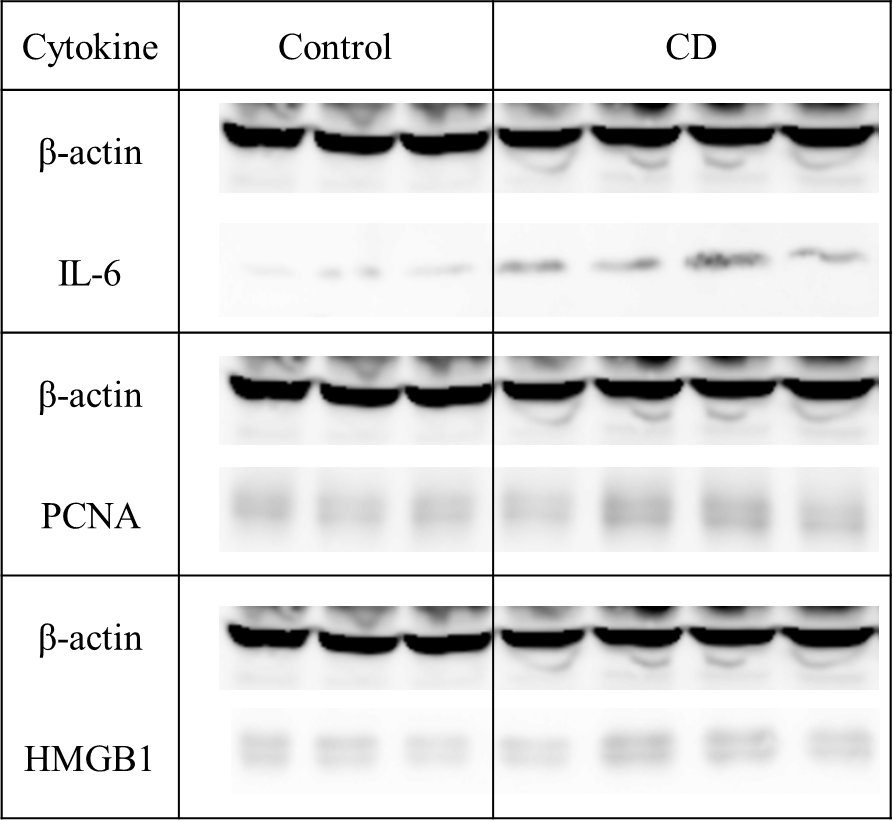


**B**


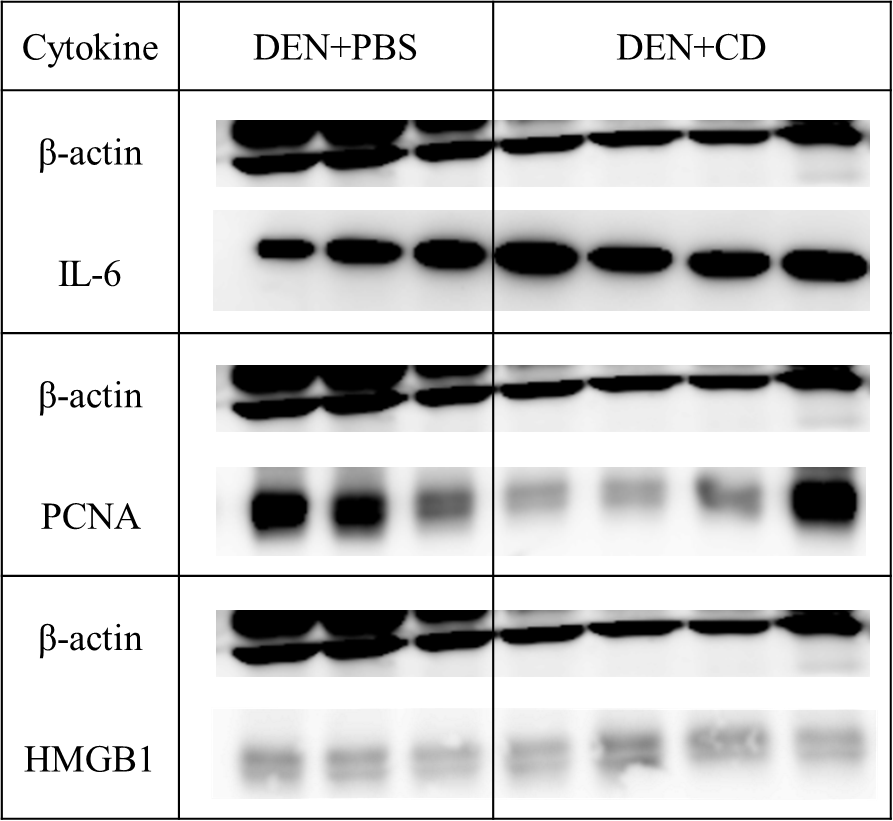


**Supplementary Figure 2.** Protein expressions of IL-6, PCNA, and HMGB1 in liver tissues of non-DEN treatment groups (A) and DEN-treatment groups (B). DEN: diethylnitrosamine.


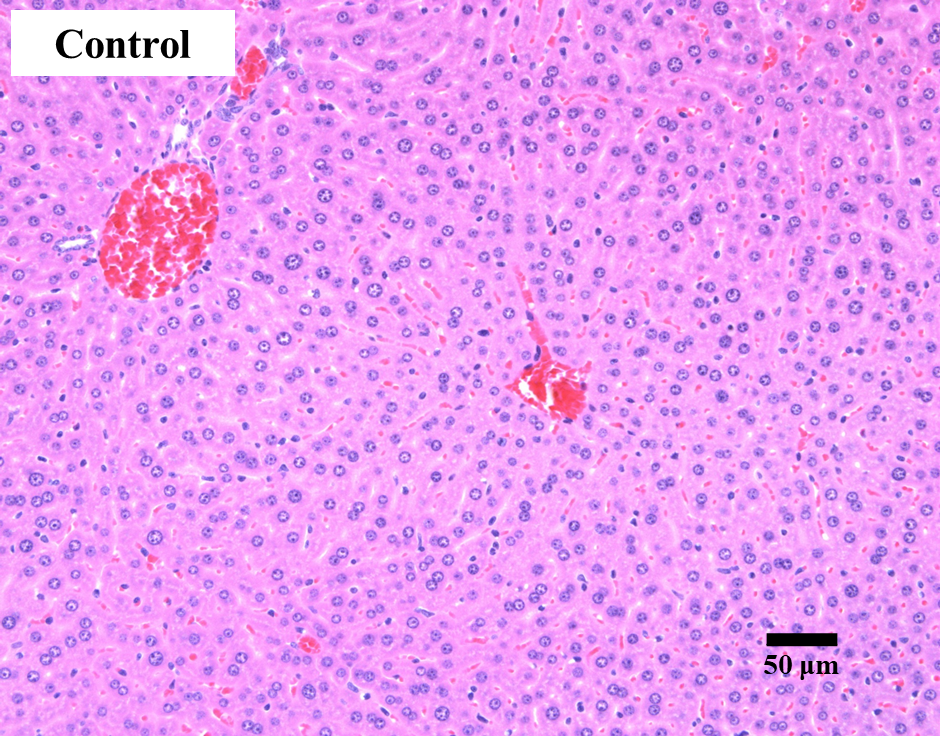


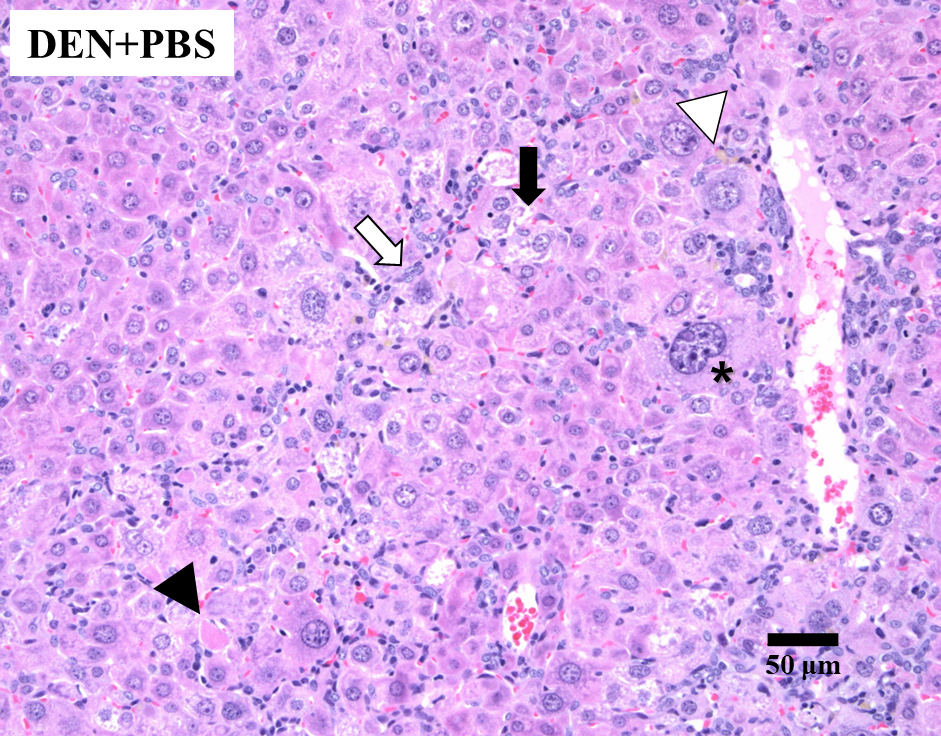


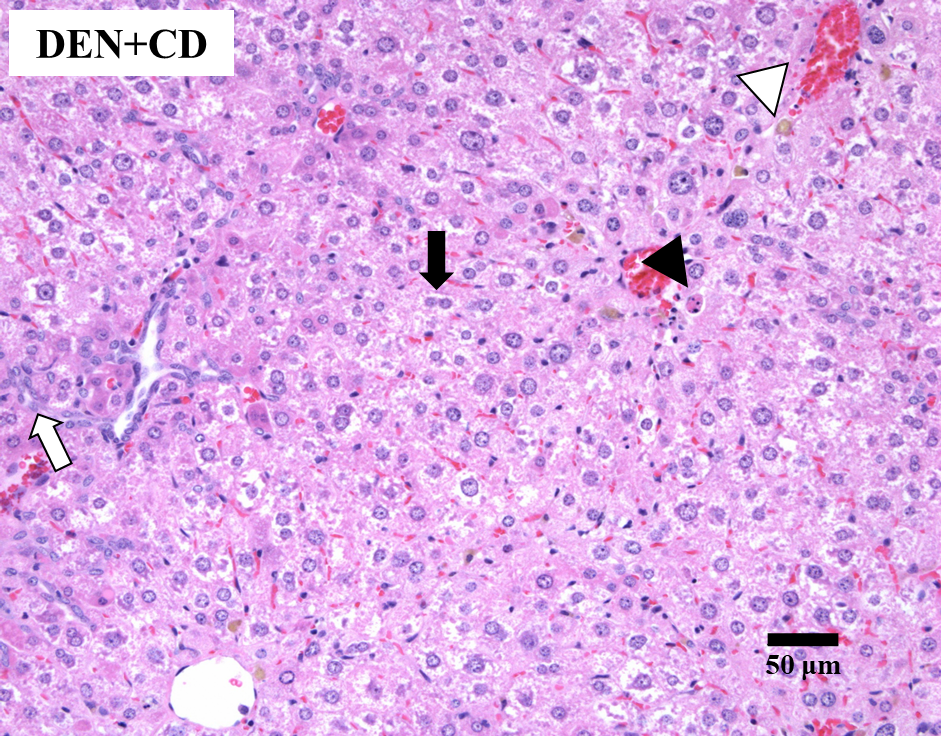


**Supplementary Figure 3.** Histopathological changes of mice liver in control and diethylnitrosamine (DEN) treatment groups (
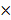
200).

Hematoxylin and eosin (H&E)-stained liver from control, DEN+PBS, and DEN+CD. Liver from control presented healthy state. In DEN+PBS, degenerated hepatocyte (black arrow), hepatocellular necrosis (black triangle), karyomegaly (star), oval cell hyperplasia (white arrow) and cholestasis (white triangle) were found. In DEN+CD, degenerated hepatocyte (black arrow), hepatocellular necrosis (black triangle), oval cell hyperplasia (white arrow), and cholestasis (white triangle) were shown. Each scale bar is indicated in the lower right corner with black line and represents 50 μm.
